# An empirical study of collaborative capacity evaluation and scheduling optimization for a CPD project

**DOI:** 10.1371/journal.pone.0200753

**Published:** 2018-08-01

**Authors:** Xiaolei Wang, Tiejun Ci, Sang-Bing Tsai, Aijun Liu, Quan Chen

**Affiliations:** 1 College of Logistics, Beijing Normal University Zhuhai, Zhuhai, China; 2 Department of Mechanical Engineering, North China Electric Power University, Baoding, China; 3 Zhongshan Institute, University of Electronic Science and Technology of China, Zhongshan, China; 4 College of Business Administration, Capital University of Economics and Business, Beijing, China; 5 School of Economics and Management, Xidian University, Xi’an, China; Southwest University, CHINA

## Abstract

In a collaborative product design project, reasonable resource allocation can shorten the development cycle and reduce cost. Team capacity evaluation and a task-team scheduling model are presented. A collaborative team capacity model is constructed, and a 2-tuple linguistic method is used to evaluate the capacity of collaborative teams. Next, the matching degree between design task and collaborative team is defined. A collaborative product design scheduling model considering task-team matching is developed. Combined with the simulated annealing operator, based on the single-coding strategy, self-adaptive multi-point cross and mutation, an improved genetic algorithm is proposed to solve the model. Finally, a case study is presented to validate the method.

## Introduction

With the increasing global competition and growing complexity of products, the division of labour is becoming increasingly specialized. As a result, the core firm requires joint development involving customers, suppliers and research institutes to overcome these limitations. Through cross-organizational collaborative product design, it can realize the maximization of resource integration and knowledge sharing as well as the improvement of design efficiency. However, in the process of collaborative product design (CPD), the diversity of design agent and interdependence and mutual restriction between tasks make the collaborative product design process quite complicated. Therefore, design task and resource should be reasonably allocated to shorten the development cycle and reduce cost.

There is a great amount of research work on the task and resource allocation of a collaborative design project. Some of these research studies focused on task identification, task relationship analysis and task scheduling based on Petri Nets[1] and Design Structure Matrix (DSM)[2–3]. Other research studies focused on the establishment of a task and resource dynamic scheduling optimization model and a model solution based on heuristic algorithm[4] and intelligent algorithm, such as the Genetic Algorithm[5], Ant Colony Optimization[6], Particle Swarm Optimization[7], Artificial Bee Colony[8]. Pang et al. [9] established a design task net and constructed a task assignment model from tasks to team members based on the principle of equilibrium-moderation. Li et al. [10] proposed a two-stage multi-agent resource allocation method, including the arbitration of manager agent and design agent selection according to task priority function. Regard collaborative production tasks as a directed weighted complex network, Yu et al. [11] proposed an evolution model for simulating collaborative production task state to perturbations. In order to deal with the collaboration between task decomposition and task scheduling, Liu et al. [12] put forward a new method for task granularity quantitative analysis, which is used to guide the coarse-grained task decomposition and recombine the subtasks with low cohesion coefficient. Currently, in the study of capacity and matching degree for CPD, Frillman et al. [13] proposed a competency model for engineers functioning in a PLM environment that emphasized individuals' competencies. Wu et al. [14] proposed a resource capability measuring method and resource capability deployment mechanism by mapping resource task capability item (RTCI) to resource physical capability item (RPCI). Combined with cost and productivity considerations, Tanuchporn et al. [15] proposed a multi-objective ergonomic workforce scheduling model to minimize the number of utilized workers and the total worker-task changeover, maximize the total worker-task fit score. Based on agent simulation, Zhang and Li [16] simulated the human working behaviours in a collaborative product development process, where the design agent selected her/his partner according to the ability and character matching degree. Furthermore, Li and Zhang [17] analysed the static single category resource scheduling problem and the multi-category resource static scheduling problem. Based on ontology and service capabilities, He and Hu [18] proposed matching rules and algorithms of manufacturing tasks and services. However, these research studies did not consider matching between tasks and the collaboration team.

For a collaborative product design project, the project is decomposed into tasks first, and then, the tasks are allocated to the collaborative team. Next, the tasks are decomposed into sub-tasks or more detailed tasks; these sub-tasks or detailed tasks are then assigned to individuals. The previous research studies have focused on the matching between a task and an individual based on task priority or designer preference. The question arises, taking the design team as a whole, from the perspective of system engineering, what is the method to realize reasonable task-team matching? Furthermore, partner selection or task assignment requires measurement of the collaborative team comprehensive capacity. This concept refers to not only individual competency but also members’ cooperation. In addition, for task allocation, it is necessary to evaluate the capability of collaborative team while considering the cost.

In the sections that follow, the capacity model of a collaborative team is presented first. Next, the 2-tuple linguistic evaluation method is adopted to evaluate the capacity of the collaborative team. Subsequently, the matching degree (MD) is defined. Afterwards, a scheduling model considering matching degree is established, and the improved genetic-annealing algorithm is designed to solve the scheduling model. An example is solved successfully to illustrate the feasibility and validity of the proposed method and model. Finally, conclusions are presented.

## Team capacity evaluation based on the 2-tuple linguistic method

### Capacity model of collaborative team

Collaborative product design, as a multi-agent and knowledge-intensive activity, emphasizes collaborative work between design teams. Moreover, creative customers and suppliers are involved. These innovative design agents have different background knowledge, experience, skill level and interests, i.e., each team has its own special abilities and resources. Therefore, collaborative product design requires not only reasonable design task decomposition but also reasonable matching between innovation team and task, such matching has important influence on the efficiency and cost of product design.

**Fig 1:**
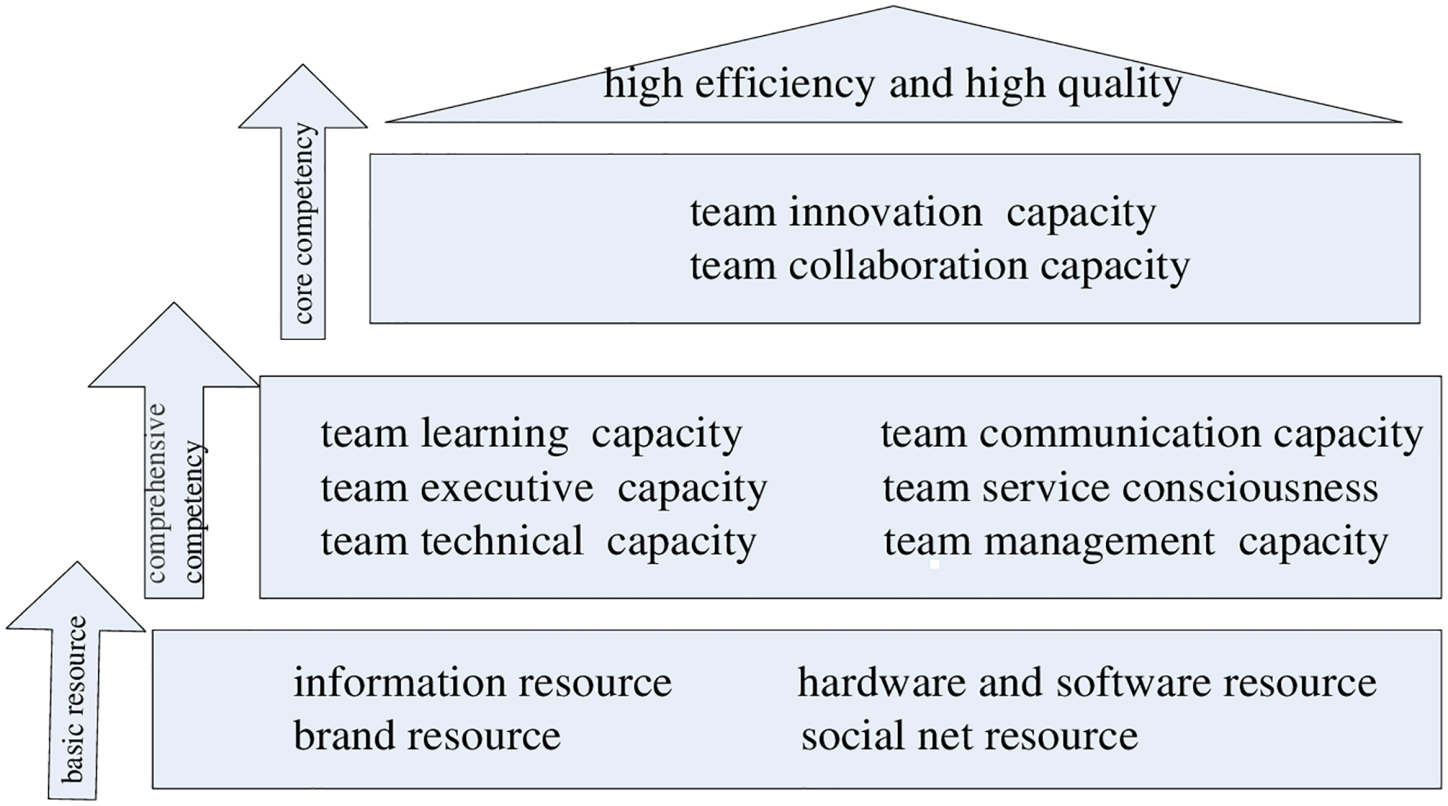
Capacity model of collaborative product design.

Capacity reflects the skill or ability sets necessary for the relevant tasks. The capacity model requires a description of the capacity elements for a task. When finding an appropriate team to conduct a design task, team capacity should be considered. For collaborative work, information sharing, goal congruence, decision synchronization, resource sharing, collaborative communication, and joint knowledge creation are significant and interconnecting elements[19–20]. Moreover, they are the prerequisite elements. Thus, the capacity model of a collaborative team is constructed as shown in Fig 1.

In the model, the basic resources of a collaborative product design team are information resources, hardware and software resource, brand resource and social net resource. The information resource includes available technical information and industry information. Important customers, government, and partners in other industries constitute the team’s social net resource. The comprehensive capacity consists of team learning capability, communication capability, team executive capability, technical capability, service consciousness, and management capability. Learning capability and communication capability are more important than the others at this level. The core capacities are team innovation capability and collaboration capability. Team collaboration requires good communication and executive ability as well as excellent team management. Learning capability and technical capability are important prerequisites and serve as the foundation for innovation. Finally, high efficiency and high quality are the ultimate goals.

### Team capacity evaluation based on the 2-tuple linguistic method

For capacity evaluation, the common methods are based on fuzzy mathematics theory, such as AHP and triangular fuzzy numbers. However, in these methods, fuzzy operation based on the extension principle increases the fuzziness of the results and causes information loss or distortion. In addition, evaluation experts often adopt natural language to express their preference, e.g., they use ‘‘high”, ‘‘average” and ‘‘low” to evaluate the team capacity, or they use ‘‘very high”, ‘‘high”, ‘‘average”, ‘‘low” and ‘‘very low” to express their evaluation results. In other words, different experts can express their evaluation information at different levels of granularity. The 2-tuple linguistic method can effectively aggregate natural language evaluation information of different levels of granularity to avoid information loss and make the result more precise[21–22]. Thus, the 2-tuple linguistic method is adopted to evaluate the competencies of the collaborative team.

The 2-tuple linguistic method represents the linguistic evaluation information by means of a two-tuple (*s*_*i*_, *α*_*i*_), where *s*_*i*_ is a linguistic label from predefined linguistic term set *S* = {*s*_0_, *s*_1_, …, *s*_*g*_}; *α*_*i*_ is the value of symbolic translation, *α*_*i*_∈[-0.5,0.5); and *g*+1 is the granularity of the set *S*. For example, a set *S* = {*s*_1_, *s*_2_, *s*_3_, *s*_4_, *s*_5_} represents the evaluation information set. The meanings of linguistic terms *s*_1_, *s*_2_, *s*_3_, *s*_4_, and *s*_5_ are ‘‘very high”, ‘‘high”, ‘‘average”, ‘‘low” and ‘‘very low”, respectively.

#### Definition 1

A real number *β*∈[0, *g*] is a number value representing the aggregation result of the linguistic symbols. The function Δ used to obtain the 2-tuple linguistic information equivalent to *β* is defined as:

$$\Delta :\left[0,\;\text{g}\right]\to \text{S}\times \left[-0.5,\;0.5\right),\;\Delta (\beta )=\left\{ \begin{array}{l} s_{k},k=round(\beta )\\ \alpha_{k}=\beta -k,\alpha_{k}\in \left[-0.5,0.5\right) \end{array}\right.$$ (1)

where round () is the rounding operator, *S*_*k*_ has the closest index label to *β*, *α*_*k*_ is the value of the symbolic translation.

In contrast, the 2-tuple linguistic variable can be converted into the crisp value *β* by the inverse function Δ^-1^:

$$\Delta^{-1}:\;\text{S}\times \left[-0.5,0.5\right)\to \left[0,\;\text{g}\right],\;\Delta^{-1}\left(s_{k},\alpha_{k}\right)=k+\alpha_{k}=\beta$$ (2)

#### Definition 2

Let *S* = {(*s*_1_, *α*_1_), (*s*_2_, *α*_2_), …, (*s*_*m*_,*α*_*m*_)} be a 2-tuple linguistic variable set at a given granularity, the arithmetic average operator of the set is computed as follows:

$$\left(\bar{s},\bar{\alpha })=\Delta \left[\frac{1}{m}\sum_{j=1}^{m}\Delta^{-1}(s_{j},\alpha_{j})\right],\;\bar{s}\in S,\bar{\alpha }\in [-0.5,0.5\right)$$ (3)

#### Definition 3

Let *S* = {(*s*_1_, *α*_1_), (*s*_2_, *α*_2_), …, (*s*_*t*_,*α*_*t*_)} be a set of 2-tuples and C = {(*c*_1_,*ß*_1_), (*c*_2_,*ß*_2_),…, (*c*_*t*_,*ß*_*t*_)} be the linguistic weighting vector of 2-tuple (*s*_*k*_, *α*_*k*_)(*k* = 1,2,…,*t*). The extended 2-tuple weighted geometric (ET-WG) operator is defined as follows[23–24]:

$$\left(\tilde{\text{s}},\tilde{\alpha })=ET\_WG_{C}(({\text{s}}_{1},\alpha_{1}),\;({\text{s}}_{2},\alpha_{2}),\ldots ,({\text{s}}_{\text{t}},\alpha_{\text{t}}))=\Delta ({\prod_{k=1}^{t}\left(\Delta^{-1}({\text{s}}_{\text{k}},\alpha_{k})\right)}^{\frac{\Delta^{-1}(c_{k},\beta_{k})}{\sum_{k=1}^{t}\Delta^{-1}(c_{k},\beta_{k})}}\right)$$ (4)

#### Definition 4

Let $\left({\tilde{\text{s}}}_{1},{\tilde{\alpha }}_{1}\right)$, $\left({\tilde{\text{s}}}_{2},{\tilde{\alpha }}_{2}\right)$, …, $\left({\tilde{\text{s}}}_{\text{u}},{\tilde{\alpha }}_{u}\right)$ be the two-tuple linguistic information with different granularities that will be aggregated. *u* is the number of groups. The improved EOWA operator is defined as:

$$\left({\text{s}}^{*},\alpha^{*})=IEOWA(({\tilde{\text{s}}}_{1},{\tilde{\alpha }}_{1}),({\tilde{\text{s}}}_{2},{\tilde{\alpha }}_{2}),\ldots ,(\tilde{{\text{s}}_{\text{u}}},{\tilde{\alpha }}_{u}))=\Delta ({\lambda^{\prime }}_{i}(\Delta^{-1}({\tilde{\text{s}}}_{i},{\tilde{\alpha }}_{i}))\right)$$ (5)

where $\left({\tilde{\text{s}}}_{i},{\tilde{\alpha }}_{i}\right)$ is the evaluation information with the *i*th maximum granularity, and ${\lambda^{\prime }}_{\text{i}}$ is the *i*th maximum number in array *λ*. *λ* = (*λ*_1_, *λ*_2_, …, *λ*_*u*_) is the weight of EOWA operator that is quantified by the fuzzy operator *E(r)*:

$$\lambda_{i}=E\left(i/u\right)-E\left(\left(i-1\right)/u\right),i=1,\;2,\ldots ,u$$

$$\text{E}(r)=\{\begin{array}{ll} 0 & r<a\\ \left(r-a)/(b-a\right) & a\leq r\leq b\\ 1 & r>b \end{array}$$ (6)

where *a*, *b*, and *r*∈[0, 1] correspond to the fuzzy linguistic quantitative principle of “half”, “most” and “as much as possible”, respectively, with the parameters (*a*, *b*) taking on values of (0, 0.5), (0.3, 0.8), and (0.5, 1), respectively.

The specific evaluation steps are as follows:

**Step 1**. The experts with the same granularity are divided into a group. The weight evaluation result of expert *k*(*k* = 1, 2,…,*t*) for capacity is denoted as ($c_{k}^{y}$, $\beta_{k}^{y}$). The evaluation result of team *j* for task *i* in capacity given by expert *k* is denoted as ($c_{kij}^{y}$, $\beta_{kij}^{y}$). According to Eq (4), the integrated information of group with the same granularity, denoted as $\left({\tilde{\text{s}}}_{ij}^{\text{y}},{\tilde{\alpha }}_{ij}^{\text{y}}\right)$, is obtained.
**Step 2**. Obtain the weight vector $\lambda^{’}=\left(\lambda_{1}^{\prime },\lambda_{2}^{\prime },\;\ldots ,\lambda_{u}^{\prime }\right)$ according to the improved EOWA operator, and then, aggregate the integrated information $\left({\tilde{\text{s}}}_{ij}^{\text{y}},{\tilde{\alpha }}_{ij}^{\text{y}}\right)$ according to Eq (5) to obtain the comprehensive evaluation information of team *j* for task *i* in capacity *y*, denoted as ($s_{ij}^{y}$, $\alpha_{ij}^{y}$). Next, the weight vector is converted into a crisp value $g_{ij}^{y}$.

## Scheduling model for CPD

### Matching degree between task and team

The matching degree refers to measure of fitness between elements. For example, when matching a project task with the collaborative team, if the matching degree is too low, then the collaborative team’s capabilities and resources are not adequate to allow them to complete the task. A higher matching degree ensures that the team can accomplish the tasks high-efficiency and high-quality, but it also means higher cost. To address this trade-off, this paper constructs a task-team matching degree model of collaborative product design project.

The task-team matching degree model is constructed in two ways: one is based on the personnel capability matching degree of collaborative team (the comprehensive capacity and core capacities in the capacity model), and the other is based on the available resources matching degree (the basic resources in the capacity model).

The matching degree between task *i* and team *j* at the dimension of personnel capabilities, denoted as *TC*_*ij*_, is defined as follows:

$$TC_{ij}=\sum_{p=1}^{8}\alpha_{i}^{p}(1\pm \sqrt{\frac{\left|{\left(g_{ij}^{p}\right)}^{2}-{\left(e_{i}^{p}\right)}^{2}\right|}{{\left(e_{i}^{p}\right)}^{2}}})$$ (7)

where *p* denotes the *p*th personnel capability, *α*_*i*_^*p*^ is the weight of the *p*th personnel capability for task *i*, $g_{ij}^{p}$ is the evaluation value of the *p*th personnel capability of team *j* for task *i*, and $e_{i}^{p}$ is the required value of the *p*th personnel capability for task *i*. In Eq (7), if $g_{ij}^{p}$>$e_{i}^{p}$, then take “+”; otherwise, take “-”.

Some available resources can be quantified, such as hardware and software. Thus, the matching degree calculation model between project task *i* and collaborative team *j* at the dimension of available resource, denoted as *TR*_*ij*_, is defined as follows:

$$TR_{ij}=\sum_{r=1}^{4}\beta_{i}^{r}*\frac{g_{ij}^{r}}{e_{i}^{r}}$$ (8)

where *r* denotes the *r*th resource, $\beta_{i}^{r}$ is the weight of the *r*th resource for task*i*, $g_{ij}^{r}$ is the available amount of the *r*th resource of team *j* for task *i*, and $e_{i}^{r}$ is the required amount of the *r*th resource for task *i*.

Furthermore, the matching degree (MD_*ij*_) between task *i* and team *j* is defined as:

$$\begin{array}{l} MD_{ij}=\omega_{i1}*TC_{ij}+\omega_{i2}*TR_{ij}\\ =\omega_{i1}(\sum_{p=1}^{8}\alpha_{i}^{p}(1\pm \sqrt{\frac{\left|{\left(g_{ij}^{p}\right)}^{2}-{\left(e_{i}^{p}\right)}^{2}\right|}{{\left(e_{i}^{p}\right)}^{2}}}))+\omega_{i2}(\sum_{r=1}^{4}\beta_{i}^{r}*\frac{g_{ij}^{r}}{e_{i}^{r}}) \end{array}$$ (9)

where *w*_*i*1_ and *w*_*i*2_ are the weights of the personnel capability and the available resource for task *i*, respectively.

### Scheduling model

In a collaborative innovation project, through rational resource selection and configuration according to the project tasks’ requirement, optimal duration and cost are achieved.

Parameters:

PT: the project duration;
*C*: the project cost;
*T*: the set of project tasks, *T* = {*T*_1_, *T*_2_,…,*T*_*m*_};
*G*: the set of collaborative teams, *G* = {*G*_1_, *G*_2_,…,*G*_*n*_}, where *n* is the number of collaborative teams;
*S* = {*s*_*t*1_, *s*_*t*2_,…,*s*_*ti*_…,*s*_*tm*_, *s*_*tm*+1_}, where *s*_*ti*_ denotes the start time of task *i*, and task *m*+1 is a virtual task;
MD_*ij*_: the matching degree between task *i* and team *j*;
*t*_*Ni*_: the standard expected execution time of task *i*;
Δ*t*_*i*_: the maximum shortened amplitude of execution time for task *i*;
*t*_*ij*_: the expected time of collaborative team *j* to execute task *i*.

For collaborative product design, the shortened duration often leads to increased costs. Chen et al. [25] proposed a linear relationship between the activity time reduction and the cost increases to transfer the time-cost trade-off problem into a linear programming problem. Thus, the optimization objective is as follows:

$$\text{min}f(x)={\text{a}}_{1}*PT+{\text{a}}_{2}*C={\text{a}}_{1}*S_{tm+1}^{}+{\text{a}}_{2}*C$$ (10)

Constraints:

$$x_{ij}=\{\begin{array}{cc} 1, & \text{team}\;\text{j}\;\text{complete}\;\text{task}\;\text{i}\\ 0 & \text{else} \end{array}$$ (11)

$$\sum_{j=1}^{n}x_{ij}=1$$ (12)

∑i∈Atxij=1 (13)

$$e_{rmin}^{i}\leq e_{r}^{i}\leq e_{rmax}^{i}$$ (14)

$$S_{t}{}_{q}=\text{max}\;\text{min}(S_{ti}+t_{ij}),T_{i}\in B_{\left(q\right)}$$ (15)

$$t_{ij}=\{\begin{array}{ll} \frac{t_{Ni}}{{\text{MD}}_{\text{ij}}}, & {\text{MD}}_{\text{ij}}\leq 1.0\\ \text{Max}\{\frac{t_{Ni}}{{\text{MD}}_{\text{ij}}},(t_{Ni}-\Delta t_{i})\}, & {\text{MD}}_{\text{ij}}>1.0 \end{array}$$ (16)

In the objective function *f*(*x*), *a*_1_ and *a*_2_ are the weights of project duration and cost, respectively. Constraint (12) expresses the resource constraint. Constraint (13) ensures that task *i* is only performed by one collaborative team. Constraint (14) ensures that one collaborative team can only perform one task at a period, *A*_*t*_ denotes the collection of tasks that are conducted at time *t*. Constraint (15) is the time constraint, and *B (q)* is the precedence activities setoff task *q*. Eq (16) is the time taken for collaborative team *j* to finish task *i* while considering the matching degree.

## The improved GA

The issue proposed in this paper is a combinatorial optimization problem. However, it is different from traditional combinatorial optimization problems because the encoding cannot be repeated. A collaborative team can execute several tasks as long as the tasks do not overlap in one period. To solve the problem, the genetic algorithm is improved, where genetic operators are used to represent the individual of feasible solution in the encoding process. Single-coding in the solution space not only eliminates the decoding process between gene space and solution space but also can enhance the accuracy and reduce the complexity of computation process.

**Fig 2:**
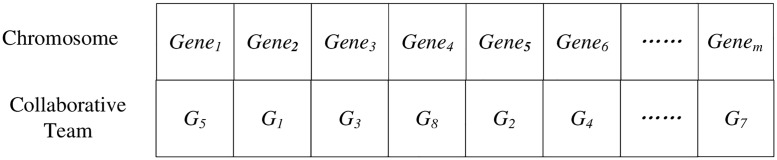
Coding.

The steps of improved genetic algorithm are as follows:

1. Coding
  Adopting decimal single coding, each gene locus represents the task code, and the number on the gene locus represents the corresponding matching collaborative team, as shown in Fig 2.
2. Fitness function
  The fitness function of GA is known as the evaluation function; it is used to determine the quality of individual. In this paper, the objective function is set as fitness function F(*x*).
  F(x)=f(x)
3. Selecting the initial population
  Randomly generate a certain number of individuals. Next, remove the repeated individuals and the individuals who do not meet the constraints, choose the best individual into the initial population and select *a*-1 individual from the remaining individuals randomly. These individuals compose an initial population with number of *a*. The probability (*p*_*i*_) that can be selected is set as follows:
  $$p_{i}=\frac{F_{i}}{\sum F_{i}}$$ (17)
4. Crossover operator
  Multi-point crossover is adopted. In the process of evolution, if the current individual fitness is lower than the average fitness, then the individual evolution is invalid. To improve the search speed, it is necessary to improve individual crossover probability. Therefore, the adaptive crossover probability strategy is adopted. The crossover probability(*p*_*c*_) is defined as
  $$p_{c}=\{\begin{array}{ccc} p_{c1} & -\frac{\left(p_{c1}-p_{c2})(F_{i}-F_{av}\right)}{\left(F_{\text{max}}-F_{av}\right)} & F_{i}\geq F_{av}\\ p_{c1} & F_{i}<F_{av} & \end{array}$$ (18)
  where *F*_*av*_ and *F*_*max*_ are the average fitness and the largest fitness, respectively.
5. Mutation operator
  Execute mutation operation for each individual, the gene changes at a certain probability and varies from 1 to *n* (*n* is the total number of collaborative team). In the process of mutation, single point mutation is used the first half of the individual, and multi-point mutation is adopted in the second part.
6. Selection operator
  The previous generation population, the population after crossover and the population after mutation constitute the selection set. Remove the individuals of the population that do not meet the constraints. Next, the best individuals of the preceding generation population, crossover population and mutation population are retained. For the remaining individuals, two individuals are selected randomly, and one of them is chosen using the simulated annealing operator with probability exp (-Δc/θ) to bring into the next generation, and the other is taken back.
  Repeat the above procedure until the number of the next generation reaches *a*, and then go to the next round.
7. Termination condition, output the optimal

When meet one of the conditions, the iteration is stopped:

1. Fitness of the best individual and the group are no longer rising;
2. The number of iterations reaches the preset number.

**Fig 3:**
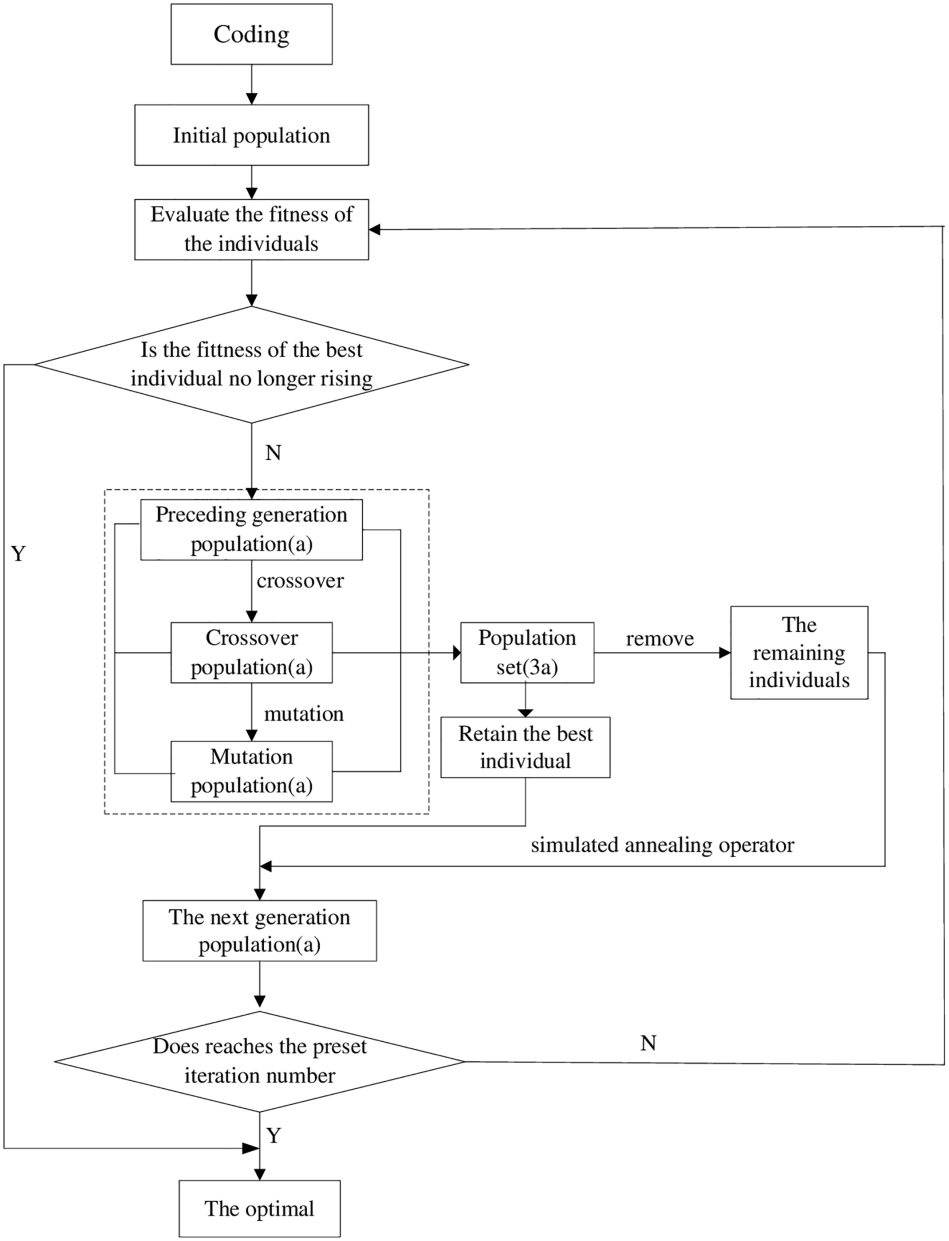
The procedure of the improved genetic algorithm.

The procedure of improved genetic algorithm is shown in Fig 3.

## Case study

**Fig 4:**
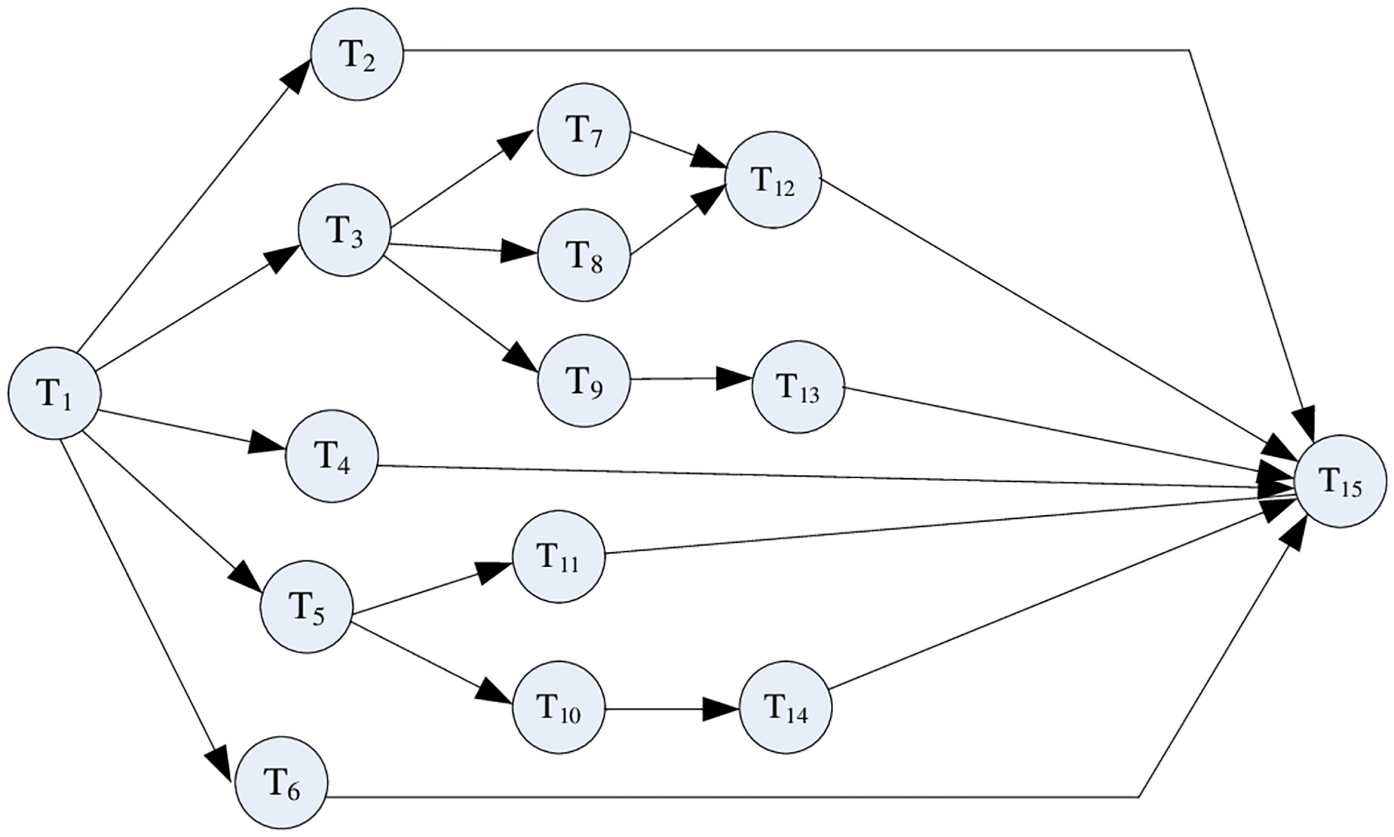
Task relationship.

First, we conducted an experiment on our scheduling optimization algorithm of mobile phone collaborative product design. The relationship of design task is shown in Fig 4. A total of 15 tasks were included in the project, and 20 collaborative teams were available.

Standard time and the maximum shorten time of the tasks are shown in Table 1.

**Table 1:** Standard execution time and the maximum shorten time of the design tasks.

	T_1_	T_2_	T_3_	T_4_	T_5_	T_6_	T_7_	T_8_	T_9_	T_10_	T_11_	T_12_	T_13_	T_14_	T_15_(Days)
*t*_*Ni*_	4	5	5	6	30	30	25	7	5	15	5	1	1	1	4
Δ*t*_*i*_	1	2	3	2	2	3	2	1	2	3	2	0.5	0.2	0.5	2

The matching degrees between the collaborative teams and the tasks are shown in Tables 2 and 3.

**Table 2:** Matching degree between collaborative teams (G_1_-G_10_) and tasks(T_1_- T_15_).

	G_1_	G_2_	G_3_	G_4_	G_5_	G_6_	G_7_	G_8_	G_9_	G_10_
T_1_	1.859	0.514	1.358	1.608	1.149	1.446	0.468	1.465	1.022	1.259
T_2_	1.604	0.911	0.725	1.422	1.719	1.209	0.665	0.570	0.541	1.131
T_3_	1.054	1.698	0.902	0.774	0.780	1.563	0.522	0.819	1.178	0.758
T_4_	1.595	0.918	0.490	1.173	1.905	1.128	1.469	1.667	1.202	0.677
T_5_	1.469	1.224	0.612	1.688	1.891	1.605	1.168	1.230	0.730	0.790
T_6_	0.862	0.630	1.155	0.680	1.472	1.150	0.920	1.589	1.688	0.829
T_7_	0.957	1.701	1.953	0.714	1.477	1.743	1.722	1.870	1.298	0.915
T_8_	1.371	0.289	0.709	1.401	1.476	0.798	1.322	0.798	0.609	0.513
T_9_	1.139	1.245	0.863	1.858	1.892	0.946	0.708	0.848	0.951	0.540
T_10_	1.822	1.995	0.660	0.860	1.039	0.918	0.694	0.980	1.501	1.213
T_11_	1.589	1.035	1.780	0.860	1.393	1.608	1.062	1.495	1.060	0.895
T_12_	0.358	0.913	0.660	0.977	0.796	0.654	1.912	0.641	1.910	0.833
T_13_	1.845	0.743	1.063	1.892	1.254	1.076	1.083	1.301	1.437	1.492
T_14_	1.049	1.282	1.588	1.251	0.759	1.644	0.556	0.411	1.160	0.919
T_15_	0.483	1.864	1.982	1.816	1.561	1.409	0.960	0.747	1.176	0.922

**Table 3:** Matching degree between collaborative teams (G_11_-G_20_) and tasks(T_1_- T_15_).

	G_11_	G_12_	G_13_	G_14_	G_15_	G_16_	G_17_	G_18_	G_19_	G_20_
T_1_	1.427	2.267	1.906	1.487	1.039	1.300	1.973	1.814	0.502	2.296
T_2_	2.244	1.852	1.338	2.211	1.360	1.882	1.902	0.879	0.852	1.745
T_3_	1.743	1.511	1.229	1.784	0.608	1.020	1.623	1.853	1.492	1.370
T_4_	1.638	1.467	0.590	1.364	0.860	2.319	1.666	1.882	0.933	1.378
T_5_	1.594	1.311	0.813	2.023	0.569	1.442	2.475	1.980	1.362	1.490
T_6_	1.291	1.004	0.930	1.351	0.555	2.383	1.208	0.748	0.644	1.674
T_7_	1.112	1.824	1.710	1.151	0.962	2.191	1.374	0.536	1.259	1.864
T_8_	2.366	1.244	0.786	1.924	0.906	1.454	1.231	1.169	1.150	1.673
T_9_	2.486	1.448	1.475	1.023	1.540	1.340	1.066	1.543	1.074	1.419
T_10_	1.499	1.385	1.805	1.150	1.168	1.919	2.175	1.746	1.387	1.364
T_11_	1.076	2.077	1.149	1.065	1.102	2.326	1.166	1.861	0.583	1.203
T_12_	1.561	1.803	1.531	1.336	1.377	1.456	2.295	1.004	0.974	1.868
T_13_	2.346	1.815	1.227	2.234	0.933	1.945	2.286	1.117	0.534	1.125
T_14_	1.568	1.638	1.658	1.367	0.644	2.193	1.052	1.502	1.528	1.285
T_15_	2.017	2.001	0.582	1.534	0.972	1.219	2.128	1.346	0.775	1.129

The task costs are listed in Tables 4 and 5.

**Table 4:** The cost that the collaborative teams (G_1_- G_10_) require to complete the task.

	G_1_	G_2_	G_3_	G_4_	G_5_	G_6_	G_7_	G_8_	G_9_	G_10_(10^4^)
T_1_	6	8	7	8	7	10	7	7	8	8
T_2_	8	6	7	6	9	9	6	5	8	7
T_3_	6	7	7	5	7	6	7	5	6	6
T_4_	8	9	10	9	11	11	10	8	10	9
T_5_	18	19	17	17	20	18	16	23	21	17
T_6_	23	26	27	27	24	25	22	20	25	24
T_7_	15	18	16	16	18	17	18	19	16	18
T_8_	12	10	10	13	13	11	14	15	14	12
T_9_	6	7	8	7	7	5	7	9	9	7
T_10_	18	17	16	21	19	18	20	19	18	17
T_11_	5	4	5	4	6	7	5	5	7	6
T_12_	2	3	4	5	2	3	5	6	4	3
T_13_	7	4	5	7	4	4	5	7	6	5
T_14_	3	5	3	4	5	6	5	6	5	4
T_15_	10	7	10	9	8	9	8	12	9	9

**Table 5:** The cost that the collaborative teams (G_11_- G_20_) require to complete the task.

	G_11_	G_12_	G_13_	G_14_	G_15_	G_16_	G_17_	G_18_	G_19_	G_20_(10^4^)
T_1_	10	7	10	8	9	10	10	8	6	10
T_2_	10	8	6	9	6	9	10	6	5	8
T_3_	9	5	6	5	7	7	9	7	5	8
T_4_	11	10	11	12	9	11	11	9	7	12
T_5_	30	18	21	19	23	24	35	20	16	24
T_6_	35	27	22	25	22	26	32	23	20	26
T_7_	16	10	16	15	18	16	16	17	19	17
T_8_	15	15	13	14	11	15	13	13	11	13
T_9_	10	8	8	9	8	6	10	8	5	7
T_10_	26	19	15	20	18	19	27	15	14	17
T_11_	6	6	5	7	5	7	6	5	4	5
T_12_	5	3	4	6	5	5	5	3	2	7
T_13_	5	7	7	4	7	7	6	7	5	6
T_14_	8	3	5	5	3	4	7	3	3	7
T_15_	15	11	11	9	10	12	17	10	8	9

The parameter configurations of the improved GA were as follows: the initial population size was 20, *P*_*c*1_ was 0.85, *P*_*c*2_ was 0.65, the mutation probability was 0.9, the maximum number of iteration was 800,*a*_1_ was 0.6, and *a*_2_ was 0.4. Based on the data above, the procedures of the improved genetic algorithm were written in Matlab and run on a PC with an Intel Core 2.4 GHz CPU, 4GB RAM, the optimal programme is shown in Table 6.

**Table 6:** Tasks—Team matching programme.

Task Number	1	2	3	4	5	6	7	8	9	10	11	12	13	14	15
Collaborative team	1	19	14	19	7	8	12	3	19	19	2	5	5	12	2

**Fig 5:**
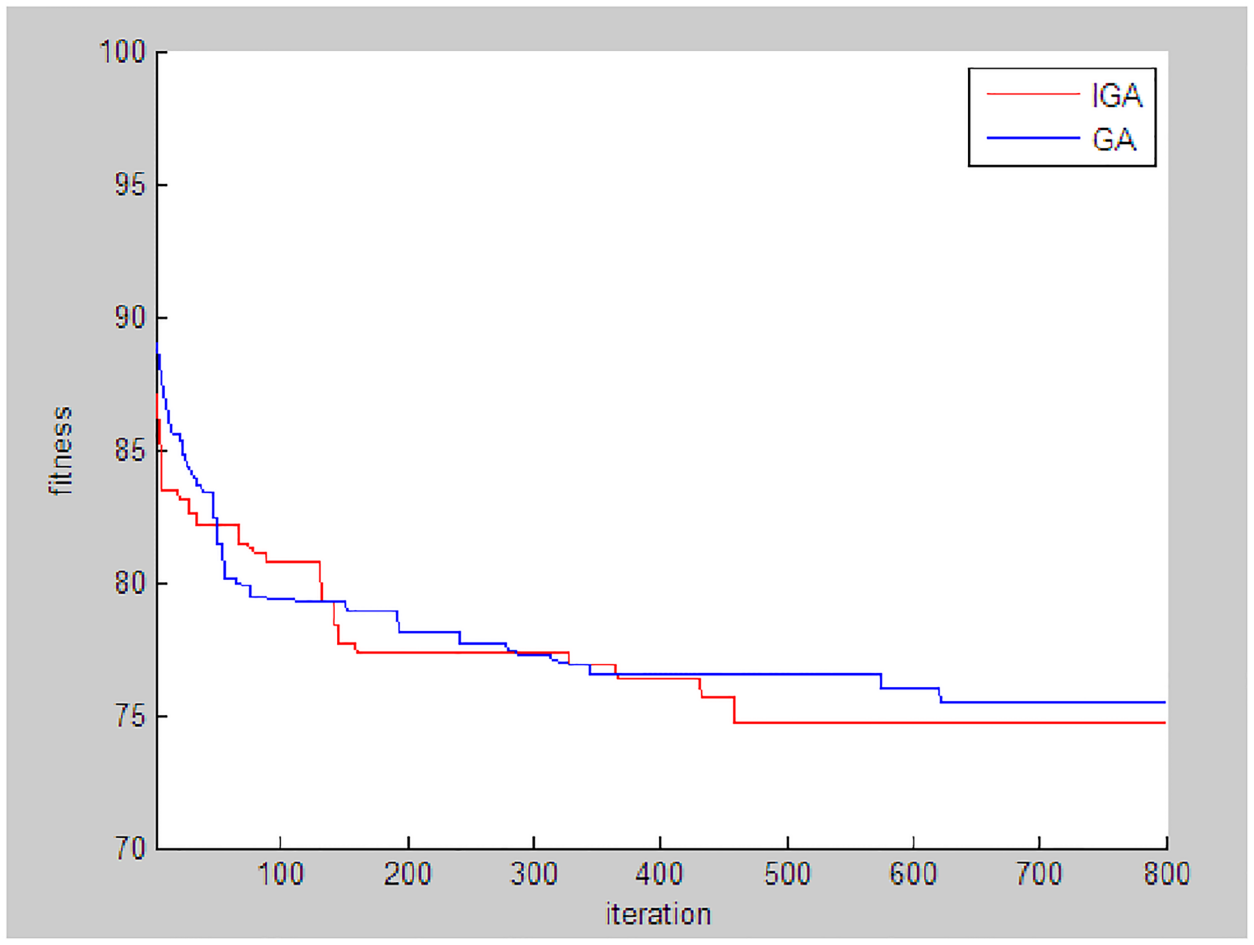
Fitness curves of the improved GA and the GA.

Under this matching programme, the objective optimal value is 74.10, the duration is 45.7days and the cost is 1,180,000 RMB. The solution obtained by GA is {1 19 4 1 7 8 12 2 19 13 2 1 5 12 2}. The fitness curve of the improved GA and that of the traditional GA are shown in Fig 5. The optimal was achieved at the 458th and the 622nd iteration by the improved and the traditional genetic algorithm, respectively. The result of the comparison revealed the advantage of the improved algorithm in finding the optimal and convergence speed, as shown in Table 7.

**Table 7:** Comparison of theimproved GA and the GA.

Algorithm	Fitness	Run time(s)	Iteration
GA	75.45	32.6	622
Improved GA	74.65	20.4	458

**Fig 6:**
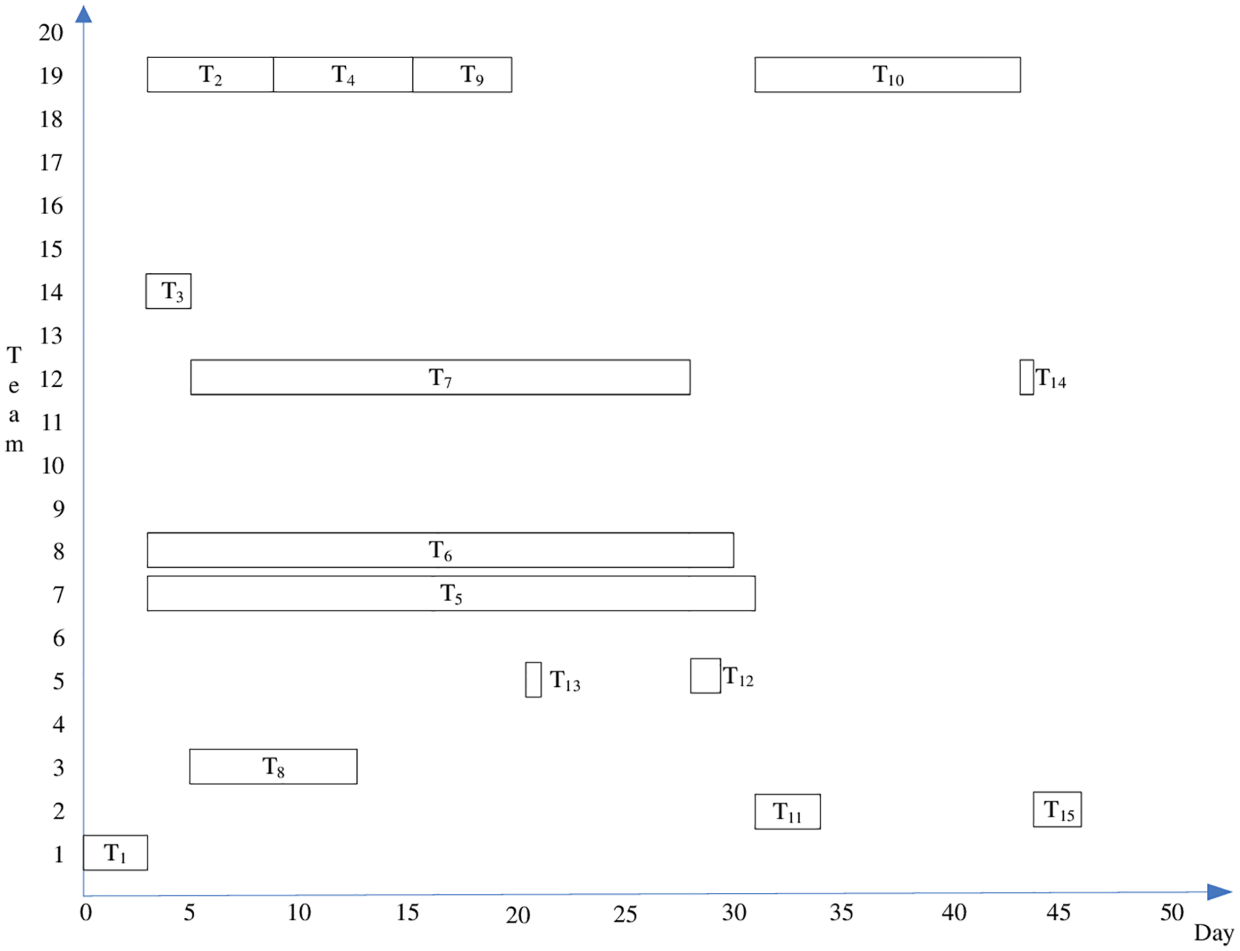
Project task allocation and timing chart.

The project task allocation and schedule plan is shown in Fig 6.

## Conclusions

In this paper, a competence evaluation and a scheduling model of collaborative product design were studied based on matching degree. In the competence model, the collaborative team capacity is composed of core competency, basic competency and basic resource. Variable competencies or resources have different effects on the matching degree. The 2-tuple linguistic method was used to avoid information loss and make the evaluation result more precise. The scheduling model considering matching degree was established considering matching degree, project duration and cost. In the improved algorithm, single-coding strategy, multi-point mutation and crossover are adopted.

Although the case study demonstrated that the proposed approach is a useful tool to obtain the reasonable programme, there are still limitations in the approach, such as the subjectivity of evaluation and the precision of resource quantization. Furthermore, during the process of collaborative product design, there may be resource conflicts and partner selection conflicts. In the future, more work on the encouragement and collaboration mechanism for collaborative design should be performed.
